# Recurrent reflex syncope in idiopathic intracranial hypertension patient resolved after lumbar puncture: pathogenetic implications

**DOI:** 10.1186/s12883-023-03451-9

**Published:** 2023-11-21

**Authors:** Roberto De Simone, Mattia Sansone, Francesco Curcio, Cinzia Valeria Russo, Gianluigi Galizia, Angelo Miele, Antonio Stornaiuolo, Andrea Piccolo, Simone Braca, Pasquale Abete

**Affiliations:** 1 Department of Neuroscience, Reproductive Sciences and Odontostomatology, Headache Centre, University Federico II of Naples, Via S. Pansini, 5, Naples, 80131 Italy; 2 Department of Translational Medical Sciences, University Federico II of Naples, Naples, Italy

**Keywords:** Syncope, Idiopathic intracranial Hypertension without papilledema, Intracranial compliance

## Abstract

**Background:**

Idiopathic intracranial hypertension is a disease characterized by increased intracranial cerebrospinal fluid volume and pressure without evidence of other intracranial pathology. Dural sinuses are rigid structures representing a privileged low-pressure intracranial compartment. Rigidity of dural sinus ensures that the large physiologic fluctuations of cerebrospinal fluid pressure associated with postural changes or to Valsalva effect cannot be transmitted to the sinus. An abnormal dural sinus collapsibility, especially when associated with various anatomical sinus narrowing, has been proposed as a key factor in the pathogenesis of idiopathic intracranial hypertension. This pathogenetic model is based on an excessive collapsibility of the dural sinuses that leads to the triggering of a self-limiting venous collapse positive feedback-loop between the cerebrospinal fluid pressure, that compresses the sinus, and the increased dural sinus pressure upstream, that reduces the cerebrospinal fluid reabsorption rate, increasing cerebrospinal fluid volume and pressure at the expense of intracranial compliance and promoting further sinus compression. Intracranial compliance is the ability of the craniospinal space to accept small volumetric increases of one of its compartments without appreciable intracranial pressure rise. In idiopathic intracranial hypertension, a condition associated with a reduced rate of CSF reabsorption leading to its volumetric expansion, a pathologically reduced IC precedes and accompanies the rise of ICP. Syncope is defined as a transient loss of consciousness due to a transient cerebral hypoperfusion characterized by rapid onset, short duration, and spontaneous complete recovery. A transient global cerebral hypoperfusion represents the final mechanism of syncope determined by cardiac output and/or total peripheral resistance decrease. There are many causes determining low cardiac output including reflex bradycardia, arrhythmias, cardiac structural disease, inadequate venous return, and chronotropic and inotropic incompetence. Typically, syncopal transient loss of consciousness is mainly referred to an extracranial mechanism triggering a decrease in cardiac output and/or total peripheral resistance. Conversely, the association of syncope with a deranged control of intracranial compliance related to cerebral venous outflow disorders has been only anecdotally reported.

**Case Presentation:**

We report on a 57-year-old woman with daily recurrent orthostatic hypotension syncope and idiopathic intracranial hypertension-related headaches, which resolved after lumbar puncture with cerebrospinal fluid subtraction.

**Conclusions:**

A novel mechanism underlying the triggering of orthostatic syncope in the presence of intracranial hypertension-dependent reduced intracranial compliance is discussed.

## Background

### Idiopathic intracranial hypertension

Idiopathic intracranial hypertension (IIH) is a disease characterized by increased intracranial volume and pressure (ICP) without evidence of other intracranial pathology. Current IIH population prevalence is 2–22/100,000 [1] but it is rapidly growing, parallel to the increase of obesity prevalence. Papilledema and chronic headache are considered IIH diagnostic landmarks although up to 15% of cases may lack headaches [2] and that the condition may present without papilledema (IIHWOP). Considered an infrequent variant of an uncommon disease itself, the actual prevalence of IIHWOP is unknown but might be much higher than currently reported due to underrating, misdiagnosis (mainly as chronic migraine) and asymptomatic cases [3]. An increased intracranial pressure associated with significant sinus stenosis can be found in about 11.1% of individuals without signs or symptoms of IIH [4]. This percentage is about 3 orders of magnitude greater than the estimated prevalence of symptomatic IIH forms with papilledema.

**Fig. 1:**
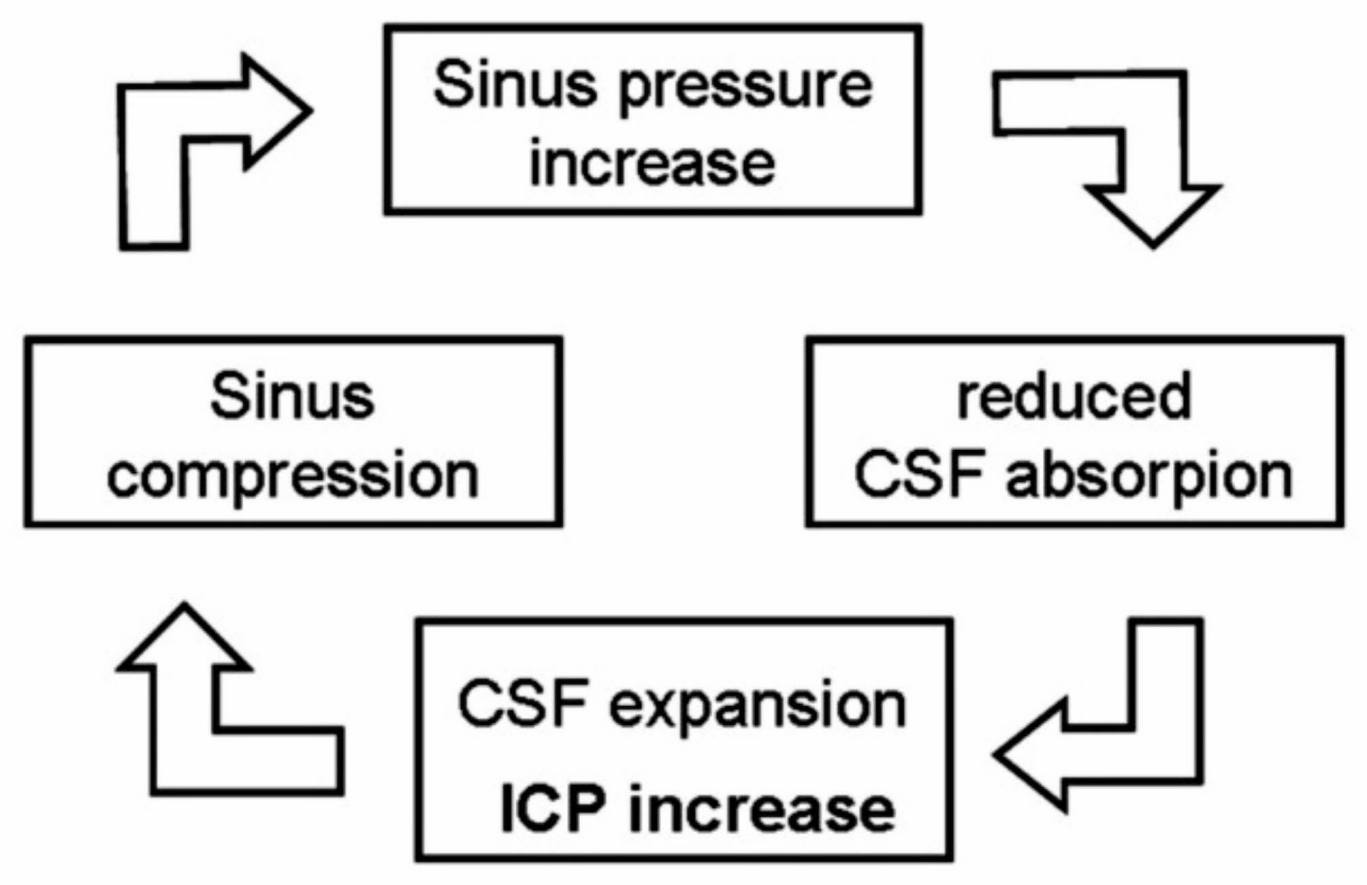
The Self-limiting Venous Collapse feedback-loop

Dural sinuses are rigid structures representing a privileged low-pressure intracranial compartment. Rigidity of dural sinus ensures that the large physiologic fluctuations of CSF pressure associated with postural changes or to Valsalva effect (under Valsalva manoeuvre up to 47 cmH_2_O [5]) cannot be transmitted to the sinus. This is a crucial constrain of brain perfusion fluid-dynamics that preserves the appropriate transmural pressure gradient required to keep the CSF reabsorption rate strictly balanced with its production level. An abnormal dural sinus collapsibility, especially when associated with various anatomical sinus narrowing (hypo/aplasia, sinus septa, giant granulations) has been proposed as a key factor in IIH/IIHWOP pathogenesis [3]. This pathogenetic model is based on excessive collapsibility of the dural sinuses that leads to the triggering of a self-limiting venous collapse (SVC) positive feedback-loop between the CSF pressure, that compresses the sinus, and the increased dural sinus pressure upstream, that reduces the CSF reabsorption rate, increasing CSF volume and pressure, promoting further sinus compression (Fig. 1). The consequent coupled rise of CSF and dural sinus pressure [6] stabilizes once the maximum sinus compression is reached. Then, a new balance between CSF and sinus blood pressure is reached, at higher pressures level [7]. It should be noted that the new balance point is reversible, at least temporarily, given an adequate perturbation is carried to any arm of the loop: sinus stenting on one side, CSF diversion or even a single CSF subtraction by LP on the other [8].

### Syncope

Syncope is defined as a transient loss of consciousness (TLOC) due to a transient cerebral hypoperfusion characterized by rapid onset, short duration, and spontaneous complete recovery [9]. The incidence of syncope is ≈ 6/1000 person-year with a significant increase after 70 years of age and a recurrence rate of 35% and 29% of physical injury [10]. Syncopal-TLOC is determined by a systolic BP drop of 50–60 mmHg at heart level (i.e., 30–45 mmHg at brain level) in the upright position with a global cerebral hypoperfusion. Global cerebral hypoperfusion represents the mechanism of syncope determined by cardiac output and/or total peripheral resistance decrease. There are many causes determining low cardiac output, including reflex bradycardia, arrhythmias, cardiac structural disease, inadequate venous return, and chronotropic and inotropic incompetence. Peripheral resistance reduction may be related to a decreased reflex activity causing vasodilatation through withdrawal of sympathetic vasoconstriction, functional and structural impairment of the autonomic nervous system, with drug-induced, primary, and secondary autonomic failure. Cardiac output and/or total peripheral resistance decrease by interacting each other identify three kinds of syncope: reflex, cardiac, and secondary to orthostatic hypotension [9].

Typically, syncopal-TLOC is mainly referred to an extracranial mechanism triggering a decrease in cardiac output and/or total peripheral resistance. Anecdotic evidence of syncope associated with significant intracranial pressure increase consequent to cerebral venous thrombosis have been reported [11, 12]. We present a case of recurrent orthostatic syncope in a patient with chronic migraine-like headache associated with IIHWOP, showing the abrupt and sustained remission of syncope episodes and headache after LP with CSF subtraction.

### Case presentation

A 57-year-old overweight woman presented with long-standing history of syncopal TLOC, which began in adolescence as sporadic (every 3–4 months) but worsened over time (up to daily). Most syncopal events occurred in standing position anticipated only by brief prodromal symptoms which included nausea, sweating and pallor. Occasionally she reported urinary incontinence related episodes. She also suffered from migraine-like headache attacks which began at the age of 23 increasing in frequency over time up to a chronic pattern (> 20 headache days per month). Headache worsened in the lying position and could present at night, awakening the patient early in the morning. Tinnitus, dizziness, sporadic vertigo attacks and occasional transient diplopia were also reported. Syncopal events were mostly independent from the presence or intensity of migraine-like pain.

**Fig. 2:**
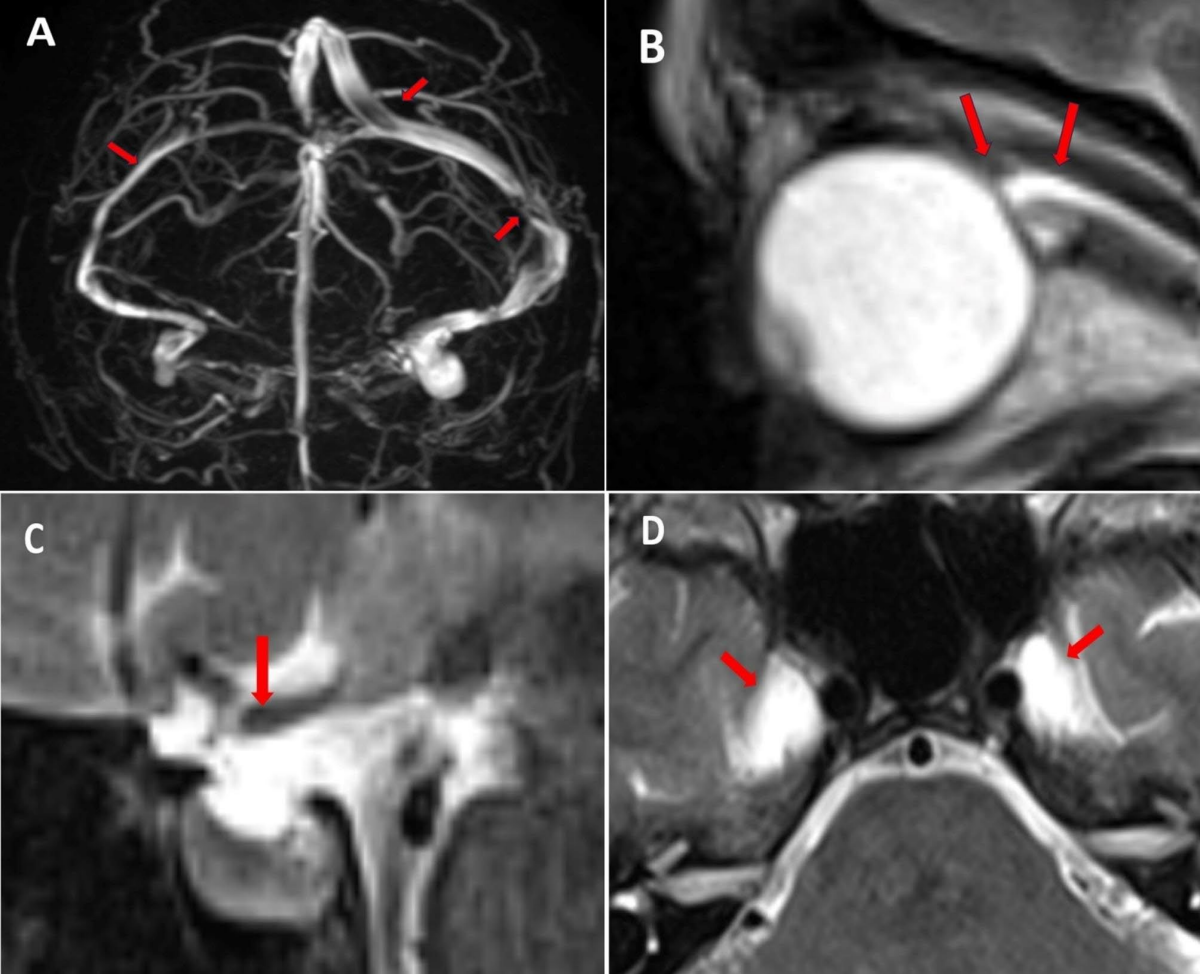
MR Venography showing (**A**) a transverse and sigmoid sinus hypoplasia on the right and two apparent flow gaps on the left at the junctions between Superior Sagittal Sinus (SSS) and left Transverse Sinus (TS) and between left TS and left Sigmoid Sinus, (**B**) ocular bulb flattening, (**C**) concave shape of the superior pituitary gland profile and (**D**) expansion of Meckel’s cave

Neurological examination was normal. Ocular fundoscopy was performed by an expert ophthalmologist, with no evidence of papilledema. Cardiac causes of syncope were excluded after extensive cardiological evaluation. Supine and standing blood pressure measurements did not show orthostatic hypotension and carotid sinus massage was negative. Tilt test was executed at admission, reporting a VASIS 2b response (cardioinhibitory response with asystole of more than three seconds) and therefore the patient was proposed for a pacemaker implantation. MR Venography (MRV) was performed, showing bilateral sinus stenosis, and, precisely, a transverse and sigmoid sinus hypoplasia on the right and two apparent flow gaps on the left at the junctions between Superior Sagittal Sinus (SSS) and left Transverse Sinus (TS) and between left TS and left Sigmoid Sinus. Apparent flow gaps are linked to a segmental flow velocity increase by focal sinus compression [13]. Brain MR showed a mild distension of optic nerve sheets associated with a defined ocular bulb flattening; dilated Meckel’s cave and a concave shape of superior profile of pituitary gland (Fig. 2A–D). We performed a lumbar puncture (LP) in recumbent position. Opening pressure (OP) was 22.3 cmH_2_O, dropping to 8 cmH_2_O after 42 ml of CSF subtraction. Therefore, a diagnosis of IIHWOP was made accordingly to the Modified Dandy Criteria [14, 15]. After the LP the patient experienced the sudden resolution of both headache and vestibular symptoms. On the day after, a new tilt test showed a VASIS 1 response (mixed-type reflex syncope), withdrawing the pacemaker indication. The patient was finally discharged at home with a diagnosis of IIHWOP associated with mixed-type reflex syncope and was commenced acetazolamide (up to 750 mg per day).

Follow-up visits during the first year confirmed the disappearance of both syncopal episodes and chronic headache. Then symptoms progressively recurred with frequent migraine-like headaches and pre-syncopal or syncopal events. Again, syncopal events were independent from presence or intensity of migraine-like pain.

A second LP, performed after 18 months, showed 20.3 cmH_2_O OP, dropping to 10.2 cmH_2_O after 36 ml CSF withdrawal. On the following day, the patient reported on the resolution of the headache and a new tilt test confirmed VASIS 1 response. At two and six-months follow-up the patient reported the absence of syncopal episodes and only sporadic migraine-like attacks.

## Discussion and conclusions

We described a case of long-standing history of recurrent syncope in the presence of sinus stenosis associated IIHWOP, which showed sustained remission after LP with CSF subtraction. After symptoms relapse, a new LP showed again good prompt response, thus suggesting a direct link between the increased intracranial pressure and the recurrent syncope. In recent years, IIH have been found in about 9% of a cohort of patients with orthostatic intolerance [16]. Interestingly, a few case-reports have demonstrated how cerebral venous thrombosis related ICP increase may lead to syncope [11, 12]. However, these cases referred to patients showing high to very high OPs. Conversely, our case presents with a CSF opening pressure of 22.3 cmH2O (and of 20.3 cm H2O after 18 months at symptoms relapse) which barely fills into the Modified Dandy Criteria, but not into the controversial Friedman criteria [17, 18] requiring mandatorily an OP greater than 25 cm H2O. This suggests that CSF pressure degree should not be seen as the key factor leading to syncope and that other related mechanisms may have a major role [8]. We speculate that intracranial compliance (IC) is the missing piece of the puzzle. In the following paragraphs, we will briefly review a likely pathophysiology.

### Intracranial compliance

The intracranial compliance (IC) is the ability of the craniospinal space to accept small volumetric increases of one of its compartments without appreciable intracranial pressure (ICP) increase. Formally it is defined as the change in CSF volume per unit of change in CSF pressure [19]. After about 10 ml of experimental CSF volume increase, the IC is almost exhausted and the ICP start to rise in an exponential manner [20]. Thus, in IIH/IIHWOP, a condition associated with a reduced rate of CSF reabsorption leading to its *volumetric* expansion, a pathologically reduced IC *precedes* and accompanies the rise of ICP. A reduced IC amplifies the physiological fluctuations of CSF pressure: large “beta waves” encompassing the upper limit of the normal ICP range promptly normalize after CSF subtraction or after medical treatment [21]. There is evidence that a reduced IC may be pathological even without a detectable increased ICP [8].

### Intracranial compliance mechanisms

Mechanisms underlying IC are not fully understood. A low-grade collapsibility of the dural sinuses is probably physiological and may contribute to IC generation by displacing some venous blood outside the cranial space. Another and probably more relevant mechanism is the displacement of CSF through the foramen magnum towards the spinal space, up to the dural sac. Indeed, the dural sac volume increases in condition of increased CSF volume and pressure, and decreases under the Valsalva manoeuvre, which by contrast raises volume and pressure of the epidural venous plexus surrounding the sac [22]. Thus, this structure is a dynamic reservoir, deeply involved in IC mechanisms, which can accommodate small volumetric changes of the intracranial compartments without ICP increase. MR studies have shown that IC is mainly generated at the spinal level [23] and normalizes after CSF subtraction by LP [24].

### An intact IC is required for brain perfusion adaptation to postural changes

**Fig. 3:**
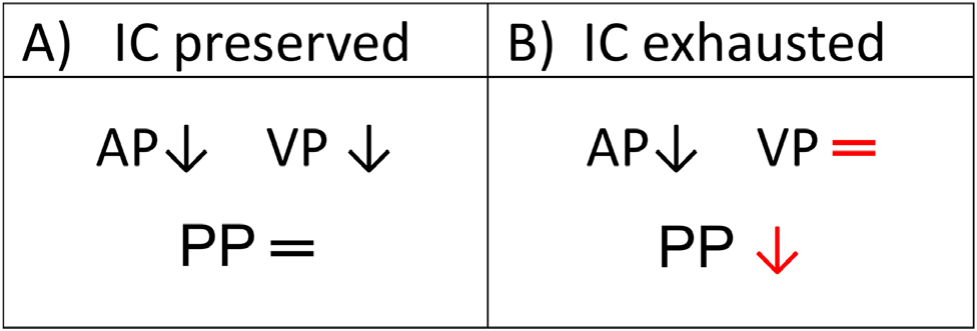
Perfusional pressure dynamics during transition to ortostathic position in conditions of preserved intracranial compliance vs. exhausted intracranial compliance. Dural sinus stenosis increases dural sinus pressure reducing CSF absorption rate and expanding the CSF volume at IC expense. (**A**) An intact IC allows the physiologic, gravity-dependent, pressure drop in cortical vein upon standing up. This compensates the correspondent arterial pressure drop and guaranties the maintaining of perfusion pressure constancy (AP-VP) despite postural changes. (**B**) A reduced IC prevents the ICP reduction and the strictly coupled cortical venous pressure drop required to balance the simultaneous gravity-dependent arterial pressure drop. This leads to the transient CPP reduction up to promote a syncope in predisposed individuals (IC: intracranial compliance; AP: arterial pressure; VP: cortical venous pressure; PP: perfusional pressure)

IC also influences the cerebral perfusion dynamics. In subjects with preserved IC, during the transition to orthostatic position, a displacement of a small amount of intracranial CSF towards the spinal space occurs physiologically. The displaced CSF volume reduces the intracranial pressure and, consequently, also the cortical vein pressure which is dynamically coupled with ICP due to the Starling resistor properties of the bridging vein (that connect physically the cortical and the dural veins, although simultaneously unpairing their pressures) [25]. Thus, during postural changes, gravity can comparably affect both, the arterial and the venous pressures of brain perfusion bed, letting be substantially unchanged their difference, namely the cerebral “perfusion pressure” (CPP) on which the cerebral flow depends. (Fig. 3)

In subjects with increased CSF volume and pressure, by definition, the IC is lacking or very low. During the transition to orthostatic position, the expanded CSF volume prevents any CSF dislocation towards the spinal cavity. Consequently, the ICP and the strictly ICP-coupled cortical vein pressure, do not decrease parallel to the arterial pressure reduction under gravity effect. This pressure mismatch temporarily reduces the CPP up to induce a syncope, likely also boosting the vasovagal reflex. Stok et al. [26] provided indeed a mathematical model of the CSF caudal shift contributing constantly to the orthostatic tolerance by its effect on CPP and, in line with it, it has been proved that a subject with a poor intracranial compliance has consequently a poor orthostatic tolerance [27]. Thus, although it is generally accepted that syncope is determined by a systolic BP drop of 30–45 mmHg at brain level, patients with IIH/IIHWOP may experience syncopal TLOC for even lower BP reduction. However, this mechanism remains immediately reversible after IC normalization by CSF removal and could explain the resolution of the daily syncopal episodes observed in our patient. Of note, the above proposed mechanism cannot occur in the presence of sufficiently rigid dural sinuses. A stiff sinus keeps its venous pressure independent from the fluctuations of CSF pressure, preserving the transmural pressure and in turn the CSF excretion rate. This ensures the constancy of the CSF *volume* and *pressure*, preserving the IC.

In summary, our case suggests that even when relatively isolated—meaning not associated with definitely pathological OP – a compromised IC hinders the displacement of a portion of cerebrospinal fluid (CSF) into the spinal canal during the transition to an orthostatic state, preventing the drop of intracranial pressure [23]. Since the intracranial pressure is dynamically coupled with cortical vein pressure due to the Starling resistor properties of the bridge vein [25] a compromised IC also prevents the expected drop in cortical veins pressure by gravity effect. This, in turn, could trigger an acute, but transient, decrease in perfusion pressure promoting syncopal events in *predisposed* individuals. The nature of such predisposition is unknown but possibly linked to a delayed compensatory cardiovascular reflex response.

## Conclusions

Intracranial compliance plays a fundamental role in cerebral perfusion homeostasis. An intact IC is required to ensure the physiologic rearrangement of brain perfusion dynamics in course of postural changes. In subjects with sinus stenosis dependent rise in dural sinus pressure, the reduced CSF reabsorption expands the CSF volume pathologically reducing the IC. This mechanism precedes the onset of a detectable increased intracranial pressure and may be isolated in some individuals, i.e. not associated with a defined CSF pressure increase. In the transition to orthostatic position, a reduced IC prevents the gravity–dependent cortical venous pressure drop while the arterial pressure physiologically decreases by gravity. The mismatch leads to a transient acute fall of CPP, eventually inducing a syncope. Considering the possible high prevalence of asymptomatic forms of sinus stenosis-associated IIHWOP [4], a pathologically reduced IC could be involved in many cases of unexplained orthostatic intolerance with or without recurrent syncope, especially in subjects with headache or other symptoms/signs of intracranial hypertension. In such patients the restoring of adequate IC may represent a new therapeutic option.

## Data Availability

All data and materials are available on reasonable request to the corresponding author.
